# Mycotoxin Uptake in Wheat — Eavesdropping *Fusarium* Presence for Priming Plant Defenses or a Trojan Horse to Weaken Them?

**DOI:** 10.3389/fpls.2021.711389

**Published:** 2021-07-26

**Authors:** Laura Righetti, Dhaka Ram Bhandari, Enrico Rolli, Sara Tortorella, Renato Bruni, Chiara Dall’Asta, Bernhard Spengler

**Affiliations:** ^1^Department of Food and Drug, University of Parma, Parma, Italy; ^2^Institute of Inorganic and Analytical Chemistry, Justus Liebig University Giessen, Giessen, Germany; ^3^Department of Chemistry, Life Sciences and Environmental Sustainability, University of Parma, Parma, Italy; ^4^Molecular Horizon srl, Perugia, Italy

**Keywords:** defense metabolites, mycotoxins, metabolomics, mass spectrometry imaging, wheat, priming plant defense

## Abstract

*Fusarium* mycotoxins represent a major threat for cereal crops and food safety. While previous investigations have described plant biotransforming properties on mycotoxins or metabolic relapses of fungal infections in plants, so far, the potential consequences of radical exposure in healthy crops are mostly unknown. Therefore, we aimed at evaluating whether the exposure to mycotoxins, deoxynivalenol (DON) and zearalenone (ZEN), at the plant-soil interface may be considered a form of biotic stress capable of inducing priming or a potential initiation of fungal attack. To address this, we used atmospheric-pressure scanning microprobe matrix-assisted laser desorption/ionization mass spectrometry imaging to investigate the activation or the inhibition of specific biosynthetic pathways and *in situ* localization of primary and secondary metabolites in wheat. According to our untargeted metabolomics investigation, the translocation of plant defense metabolites (i.e., hydroxycinnamic acid amide and flavones) follows the mycotoxin accumulation organs, which is the root for ZEN-treated plantlet and culm for DON-treated sample, suggesting a local “defense-on-demand response.” Therefore, it can be hypothesized that DON and ZEN are involved in the eavesdropping of *Fusarium* presence in soil and that wheat response based on secondary metabolites may operate on multiple organs with a potential interplay that involves masked mycotoxins.

## Introduction

*Fusarium* mycotoxins represent a major threat for cereal crops and food safety. Since the early stages of infection and few days after anthesis mostly, these substances are inoculated in plant cells of spikelets tissues by fungal hyphae. Once distributed in infected plants, mycotoxins may undergo various enzymatic biotransformations, translocations, and compartmentations to reduce toxic effects and increase disposability, generating compounds usually described as masked mycotoxins (Berthiller et al., 2013).

Despite their overt role in plant disease, mycotoxins may also be present in soil in minor amounts, potentially determining an environmental exposure in species with shallow roots like wheat (Champeil et al., 2004; Bucheli et al., 2008; Elmholt, 2008; Hartmann et al., 2008). Root uptake of mycotoxins from the soil and their translocation to above-ground organs has been demonstrated in a growing range of disease-free crops including wheat, both under field and *in vitro* conditions (Hariprasad et al., 2013; Snigdha et al., 2015; Rolli et al., 2018). In most occasions, biotransformation and a selective accumulation of both parent compounds and masked forms were reported in different above and below-ground organs (Meng-Reiterer et al., 2016; Righetti et al., 2020). This phenomenon is not uncommon but instead shared with other natural and human-made organic compounds, including allelopathic substances and pharmaceuticals (Tanoue et al., 2012; Bártíková et al., 2015). Along with this defensive strategy, the absorption of those xenobiotics is known to elicit multiple biochemical responses in the recipient plants, ranging from phytotoxic effects to an increased metabolic activity aimed at managing the related stress (D’Abrosca et al., 2013; Scognamiglio et al., 2014). For instance, in case of allelopathic compounds, the root uptake by their target species represents a blueprint for weakening rivals and increasing success in a competitive environment (Mortensen et al., 2003; Hartmann et al., 2008; Owens et al., 2013; Aslam et al., 2017). At the same time, some organic substances available at the root-soil interface may prime an increased biosynthesis of secondary metabolites involved in the inducible defense, thus acting as an early alert system for plants whose efficiency is often related to pathogen resistance (Conrath et al., 2002; Tenenboim and Brotman, 2016; Alagna et al., 2020).

While previous investigations have described plant biotransforming properties on mycotoxins or metabolic relapses of ongoing fungal infections in plants (Mandala et al., 2019), so far, the potential consequences of radical exposure in healthy crops are mostly unknown. Wheat metabolome, for instance, undergoes some changes both during *Fusarium* infection and following direct injections of trichothecenes in spikes, including a broad defense response mediated by secondary metabolites (Kazan and Gardiner, 2018). On this regard, a putative protective role of phenylpropanoids, flavonoids, hydroxycinnamic acid amides, phenolics, and other antioxidant metabolites in restraining the growth of *Fusarium* spp. is recently emerging. This includes the potential inhibition of toxin biosynthesis at the transcriptional level and possible increased resistance to the pathogen employing a combination of cell wall strengthening, phytoalexin biosynthesis, and increased radical scavenging (Warth et al., 2015; Atanasova-Penichon et al., 2016; Gauthier et al., 2016; Bilska et al., 2018; Schöneberg et al., 2018; Doppler et al., 2019). Such behavior may be related to the mechanism underlying the toxicity of some trichothecenes, which encompass an inhibition of protein synthesis but also intense oxidative stress (Audenaert et al., 2013).

In the environment, plants need to cope with a variety of biotic pressures simultaneously. Thus, they have evolved a complex array of systems to sense, detect, and react to pathogens and competitors (Tenenboim and Brotman, 2016). Simultaneously, the evolution has tuned in fungi and bacteria a biochemical arsenal capable of weakening the responses mentioned above and overcoming plant defenses. Many of these communications and the cascade of effects they activate (or inhibit) are mediated by secondary metabolites that may act as primers or as disruptors, as an advanced alerting system or a virulence factor (Martinez-Medina et al., 2016). These effects may often coexist in the same molecule, albeit they may vary according to the entity of the exposure.

In this regard, the recently discovered capability of disease-free wheat to absorb mycotoxins such as deoxynivalenol (DON) and zearalenone (ZEN) through roots, requires elaborating on their hypothetical ecological functions (Rolli et al., 2018; Righetti et al., 2020). One question may concern the understanding of eventual plant stress responses elicited by mycotoxins at the root-soil interface. DON is a known virulence factor, but it is unclear if it may also play the same role during minimal soil exposure in healthy plants (Audenaert et al., 2013). ZEN is instead considered capable of hormone-like effects in plants, but it must be understood if and how this may affect the secondary metabolism of healthy wheat following root absorption (Biesaga-Kościelniak and Filek, 2010). From a larger perspective, some evidence is needed to clarify if root uptake of mycotoxins in wheat is more likely to prime inducible defense responses, if it may ease the colonization by *Fusarium*, or if it has no relevant consequences on plant metabolism.

The gradual probing of such inquiries is nowadays eased by the availability of analytical methods allowing to operate under the perspective of system biology. In this regard, modern metabolomics coupled with mass spectrometry imaging (MSI) may allow a proper insight into the interplay between plants and mycotoxins, highlighting potential variations and correlations between multiple metabolites. Atmospheric-pressure scanning microprobe matrix-assisted laser desorption/ionization (AP-SMALDI) MSI, for instance, is versatile enough to cover a broad range of plant metabolites, allowing simultaneous localization clues about the putative activation and the spatial, histological distribution of specific metabolic pathways (Bhandari et al., 2015, 2018; Sturtevant et al., 2016; Tenenboim and Brotman, 2016).

The present study aims to evaluate whether the exposure to mycotoxins at the plant-soil interface may be considered a form of biotic stress capable of inducing priming or a potential initiation of fungal attack. To do so, we used AP-SMALDI MSI that enable to localize *in situ* the activation or the inhibition of specific biosynthetic pathways of primary and secondary metabolites following mycotoxins treatment. An approach based on a recently validated and highly standardized *in vitro* system based on micropropagated wheat plantlets, was selected to limit confounding factors due to environment, culture, genetics, and developmental stage (Righetti et al., 2020). Given the known translocation to most mycotoxins’ above-ground organs and their masked forms, and considering that priming can act at a distance, the effects on both roots and culm were monitored.

## Materials and Methods

### Chemicals and Reagents

Analytical standards of DON (100 μg mL^–1^ in acetonitrile), deoxynivalenol-3-glucoside (DON-3Glc) (50.6 μg mL^–1^ in acetonitrile) ZEN (100 μg mL^–1^ in acetonitrile), α-ZEL (10 μg mL^–1^ in acetonitrile) and β-ZEL (10 μg mL^–1^ in acetonitrile) were obtained from Romer Labs (Tulln, Austria). Zearalenone-14-glucoside (ZEN14Glc) was synthesized and purified in our laboratory. Zearalenone-16-glucoside (ZEN16Glc) was kindly provided by Prof. Franz Berthiller (IFA-Tulln, University of Natural Resources and Life Science, Vienna).

2,5-dihydroxybenzoic acid (DHB), α-cyano-4-hydroxycinnamic acid (CHCA), 9-aminoacridine (9AA), *para*-nitroaniline (pNA), Girard T reagent, hydrazine hydrate, trifluoroacetic acid (TFA), bidistilled water, MS grade acetone and acetonitrile were purchased from Sigma-Aldrich (Steinheim). The gelatin used for the embedding was obtained from VWR International (Darmstadt, Germany). Glass microscope slides (ground edges, super frost) were obtained from R. Langenbrinck (Emmendingen, Germany).

### Plant Material and Growth Conditions

*Triticum durum* seeds (Kofa variety) were germinated to obtain plantlets, as previously reported (Rolli et al., 2018; Righetti et al., 2020). Briefly, plants were placed for 14 days in a glass jar containing sterile Murashige and Skoog medium spiked with 100 μg of DON and ZEN in two separate experiments. The experiment time span was previously optimized to avoid visual symptoms in the control experiments (i.e., leaf senescence). Liquid medium without mycotoxin was used in all experiments as a control. All the experiments were carried out in triplicate. After sampling, segments of 20 mm of roots and culms were obtained by transverse cuts with a scalpel blade.

Quantitative analysis of DON and ZEN and their metabolites (whose analytical standards were available) in both plant material and growing media is described in Supplementary Information Note 1.

### Evaluation of MALDI Matrices for Mycotoxins

The dried-droplet method was initially used to assess five matrices and different combinations of solvents by mixing 1 μL of mycotoxins standard with 1 μL of matrix solution and spotting 0.5 μL onto an 80-well stainless-steel plate.

### Sample Preparation

Sample preparation for MALDI was performed following the protocol previously optimized (Bhandari et al., 2015). Briefly, fresh samples were embedded in 2% (w/v) gelatin solution in a cryo-mold, and then 20 μm thick sections were cut at –20°C using a cryomicrotome (HM525 cryostat, Thermo Scientific, Dreiech, Germany). The sections were transferred to a glass slide and kept at –80°C until the day of the analysis. Before the matrix application, optical images of the sections were captured using a digital microscope VHX-5000 (Keyence GmbH, Neu-Isenburg, Germany). DHB (30 mg mL^–1^) in acetone:water (50:50, v/v, 0,1% TFA) was chosen as a matrix for untargeted metabolomics purpose and sprayed using an automated pneumatic sprayer system (SMALDIPrep, TransMIT GmbH, Giessen, Germany) (Bouschen et al., 2010) to ensure uniform coating of tissue sections with the microcrystalline matrix. The size and uniformity of the deposited crystals were checked prior to AP-SMALDI MS imaging experiments. At least two biological replicates of each tissue (i.e., root and culm) were analyzed by MSI.

### AP-SMALDI MS Imaging Analysis

Plant tissue sections imaging experiments were performed using a high spatial resolution (≥ 5 μm step size) atmospheric-pressure scanning microprobe matrix-assisted laser desorption/ionization ion source (AP-SMALDI5 AF, TransMIT GmbH, Giessen, Germany) coupled to a Q Exactive HF orbital trapping mass spectrometer (Thermo Fisher Scientific GmbH, Bremen, Germany). The minimum laser beam focus results in an ablation spot diameter of 5 μm (Römpp and Spengler, 2013). For the experiments described below, a laser step size of 7–20 μm was set, depending on the plant organ under investigation. The mass spectrometer was operated in positive-ion mode. The following parameters were set: scan range *m/z* 250–1000*;* spray voltage + 3 kV; capillary temperature 250°C, automatic gain control (AGC) was disabled; cycle time for one pixel 1 s. Internal mass calibration was performed using known matrix ion signals as lock mass values (*m/z* 716.12461), providing a mass accuracy of better than 2 ppm root mean square error over the entire measurement.

### Data Processing and Image Generation

Raw data (imzML format) were imported and processed using the LipostarMSI software package (Tortorella et al., 2020). Ion images of selected *m/z* values were generated with a bin width of ±2 ppm and normalized to total ion current (TIC). To better visualize the localization of selected metabolites, TIC images for each dataset were calculated and used to semi-automatically (using two arbitrary teaching points on the high resolution and TIC image) co-register with corresponding high-resolution images. Metabolite annotation of MSI data was performed against publicly available LIPID MAPS and PlantCyc databases by accurate *m/z* matching, isotopic pattern and chaos score within user-set tolerances. Metabolite identification is reported following the guidelines proposed by Schymanski et al. (2014).

Data in imzML format were deposited in the METASPACE platform^1^ (Palmer et al., 2017).

## Results

### DON and ZEN Uptake

In previous studies (Rolli et al., 2018; Righetti et al., 2020), we demonstrated that wheat plantlets could absorb and translocate ZEN, DON, and their masked forms from roots to above-ground organs. As this phenomenon occurs in disease-free wheat, metabolic profiling assisted by *in situ* localization may offer insights regarding these substances’ potential role in weakening or strengthening plant inducible defense.

**FIGURE 1:**
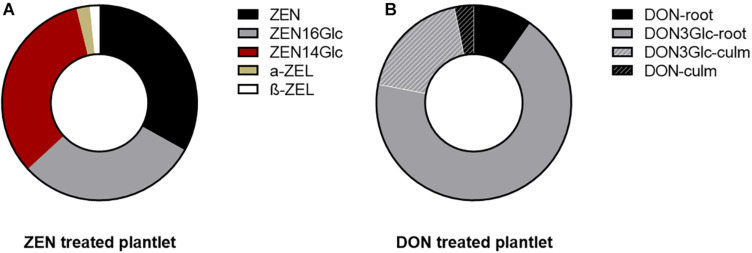
Pie chart representing the mycotoxin biotransformation and distribution in the root (for ZEN) and in the whole plantlets (for DON). Each ring represents the total portion of parent toxin and its derivatives in the plantlets, considering the sum of signals quantified in roots and culm. **(A)** ZEN and its phase I and phase II metabolites quantified in the root. Their above-ground accumulation was below the limit of detection. **(B)** DON underwent biotransformation to a large extent into its glycosidic form. Fill pattern sections of the ring represent the portion of DON and DON3Glc translocated to the above-ground organs.

The evidence mentioned above was once again confirmed, highlighting a different intensity. However, as ZEN was almost completely absorbed after 14 days, the average residual DON in the medium was about 86% (see Supplementary Figure 1). In both cases, 100 μg mL^–1^ of mycotoxin did not induce evident symptoms of phytotoxicity. The remarkable difference might be ascribable to their different n-octanol-water partitioning coefficients (log Pow of 3.6 and –0.71 for ZEN and DON, respectively), suggesting an easier passive absorption of ZEN through the waxy surface of roots (Chiou et al., 1998). Such behavior is symmetrical to the one described for most xenobiotics of human origin. The uptake is positively correlated with log Pow and likely driven by chemical adsorption onto the root surface. Once absorbed from the media, both ZEN and DON underwent biotransformation at a large extent. As previously reported (Rolli et al., 2018), after 14 days from initial exposure, 23-33% of quantified ZEN was found exclusively in roots, while the remaining was mostly conjugated to ZEN16Glc and ZEN14Glc or reduced in minimal amounts to α- or β-ZEL (see Figure 1A). Intense biotransformation but also remarkable root accumulation for ZEN and its derivatives were observed without evidencing a consistent translocation to epigean organs.

On the contrary, 20% of the absorbed DON (sum of DON and DON3Glc, measured as relative abundance) was found translocated to leaves and culm. In comparison, the remaining 80% was segregated in roots. The parent DON accounted only for 13%. Simultaneously, the majority was metabolized into the less toxic form DON3Glc, as summarized in Figure 1B. The high DON-to-DON3Glc conversion observed is consistent with previous results on the high detoxification ability of Kofa durum wheat line (Cirlini et al., 2013).

### MALDI Matrix Optimization

To properly locate mycotoxins in tissues and relate their accumulation to plant metabolite biosynthesis, we optimized ionization and detection parameters for ZEN and DON.

Five matrices (DHB, CHCA, 9AA, pNA and hydrazine hydrate) were tested with the DON and ZEN standards. However, when performing on-tissue MSI experiments with these matrices, ZEN and DON were not detected. Further details on the matrices are summarized in Supplementary Information Note 2 and Supplementary Table 1.

Therefore, DHB was chosen as the matrix for the MALDI-MSI experiment of wheat samples, which is appropriate for detecting plant metabolites at high sensitivity in positive-ion mode (Bjarnholt et al., 2014; Bhandari et al., 2015) and thus suitable also to detect metabolomic changes in response to mycotoxins accumulation. As demonstrated in previous literature (Berisha et al., 2014; Bhandari et al., 2015, 2018; Hickert et al., 2016), the ionization of this class of molecules by MSI is quite challenging especially for low molecular weight mycotoxins (<600 Dalton). As most trichothecenes, DON was never detected using MALDI-MSI, most likely due to the low ionization efficiency and its usual concentration at trace level in tissues (Berisha et al., 2014; Bhandari et al., 2015). The only detection of DON on the surface of wheat grain was reported by Berisha et al. (2014) using laser desorption followed by electrospray postionization (LD-ESI) MSI.

On the other hand, higher molecular weight mycotoxins such as fumonisins (Hickert et al., 2016), enniatin and beauvericin (Bhandari et al., 2018) were nicely detected in MALDI imaging experiments in maize kernels with *Fusarium verticillioides* and wheat stem infected with *Fusarium graminearum*, respectively. Such a scenario suggests the importance of screening and development of new MALDI matrices to improve the ionization efficiency for a variety of natural toxins and small plant metabolites.

### Untargeted Metabolomics

Given the different effects of ZEN and DON in plants and their diverse biotransformation and distribution in wheat tissues, two distinct patterns were expected in terms of metabolic response. Root and culm samples were, therefore, independently processed. Substances differentially expressed in treated and control samples are listed in Supplementary Tables 2, 3. In contrast, those discussed throughout the manuscript are listed in Table 1. Further analytical details on accurate mass, detected adduct, formula and error ppm are summarized in Supplementary Table 4. It must be highlighted that most reported secondary metabolites were increased in treated samples, and no significant inhibition of biosynthesis was noticed. Only a few dipeptides were found in higher amounts in control samples. At least in terms of secondary metabolism, this would suggest an enhancement of induced defense rather than its impairment.

**TABLE 1:** Differentially accumulated metabolites in response to DON and ZEN treatment, whose *m/z* images are reported in the manuscript (qualitative abundance are reported as: N.D, not detected; +, detected with low relative abundance; ++, detected with high relative abundance).

Class	Compounds	Localization (treatment)	Control	ZEN	DON
Amino acids, peptides, and analogs	Tryptophyl-alanine	Root cortex and pith	++	N.D.	N.D.
Glycerophospholipid	PC (34:3)	Root cortex	N.D.	++	++
Galactolipid	DGDG (34:2)	Root pith	N.D.	++	++
Hydroxycinnamic acids amides	Feruloylagmatine	Root cortex (ZEN)/Leaf sheath (DON)	N.D.	++	++
Flavonoid glycosides	Kaempferol-rhamnoside-glucoside	Root cortex (ZEN)/Leaf sheath (DON)	N.D.	++	++
Diacylglycerols	DG (42:5)	Root endodermis	+	+	+
Hydroxycinnamic acids amides	Coumaroylagmatine	Root cortex (ZEN)/Leaf sheath (DON)	N.D.	++	++
Glycerophospholipid	Glycerophosphocholine	Epidermis and vascular bundles	N.D.	++	++
Flavonoid glycosides	Patuletin diglucoside	Stem pith	N.D.	N.D.	++
Pteridines and derivatives	Dihydropteroic acid	Leaf sheath	N.D.	N.D.	++

*Classes have been taken from LIPID MAPS and PlantCyc databases.*

#### Untargeted Metabolomics – Effects on Roots

**FIGURE 2:**
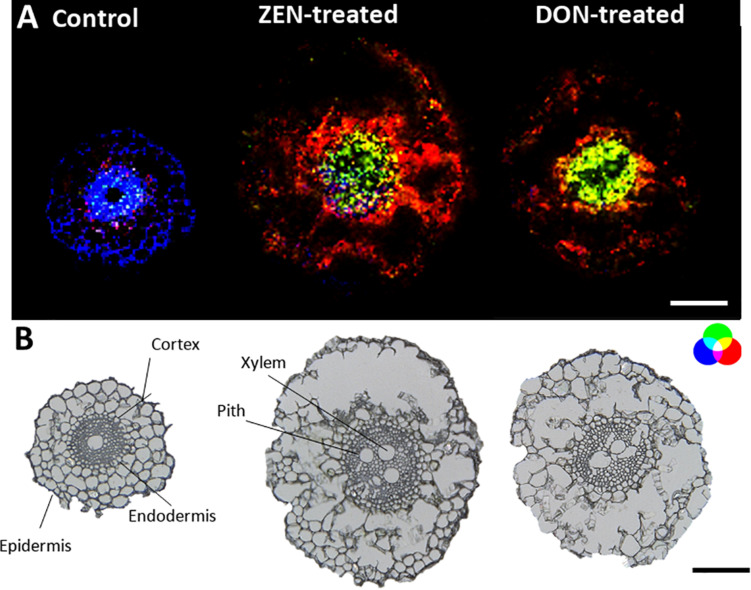
Tissue-specific changes induced in the wheat root by mycotoxins accumulation. **(A)** RGB overlay of *m/z* images of PC (34:3) [M + K]^+^, *m/z* 794.5099 (red), and DGDG (34:2) [M + K]^+^ *m/z* 955.5717 (green) accumulated in the cortex cells and in the central stele of treated samples, respectively. Distribution of tryptophyl-alanine, [M + NH_4_]^+^ *m/z* 293.1608 (blue), exclusively located in the control root. **(B)** Optical images of roots with major morphological features labeled. The structural integrity of some roots was damaged due to the fragility of the tissue. The large vacuole in the root cell caused the cells to rupture due to the temperature change during the thawing process. AP-SMALDI MS images were obtained at 7 μm imaging step size and normalized to the total ion current on a 0-60% intensity scale. Scale bars: 200 μm.

Roots MS imaging measurement describes the localization of phosphatidylcholine (PC) (34:3) in the root cortex and digalactosyl-diacylglycerol (DGDG) (34:2) accumulated in the central stele of mycotoxin-treated wheat samples (Figure 2A), representing the enhanced defensive state of root against both biotic stress and mycotoxin exposure during infection (Perlikowski et al., 2016; Rubert et al., 2017). This accumulation appears to be the consequence of a non-specific systemically induced resistance, with the same trend in both DON- and ZEN- treated roots. The opposite tendency was observed for some peptides, including tryptophyl-alanine (blue) that was mapped only in the cortex cells and the central stele of the control roots, with a specific absence from the metaxylem vessel.

Apart from PC (34:3), other glycerophospholipids (see Supplementary Table 2) were detected in mycotoxin-treated roots compared to the control. PCs were found to be localized in the cortex cells, while phosphatidic acid (PA) was mainly localized in the pith of treated roots.

**FIGURE 3:**
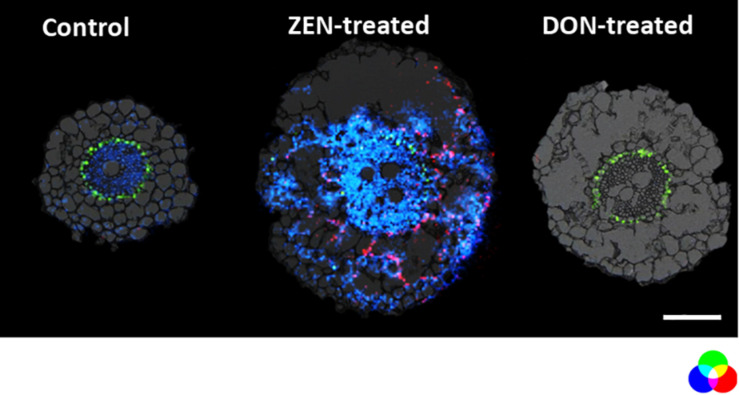
Distribution of metabolites representing the enhanced defensive state of root organ triggered by DON or ZEN treatment. RGB overlay of *m/z* images of DAG (42:5) [M + K]^+^ *m/z* 737.5481 (green), feruloylagmatine [M + H]^+^ *m/z* 307.1765 (blue), kaempferol-rhamnoside-glucoside [M + Na]^+^ *m/z* 617.1478 (red) with the optical images. The distribution of DAG (42:5) (green) is homogeneous regardless of the treatment. Its ion image is well correlated with the optical image in the endodermis. The AP-SMALDI MS images were obtained at 7 μm imaging step size and normalized to the total ion current on a 0-60% intensity scale for feruloylagmatine (blue) and kaempferol-rhamnoside-glucoside (red) and 0-40% intensity scale for DAG (42:5). Scale bar: 200 μm.

**FIGURE 4:**
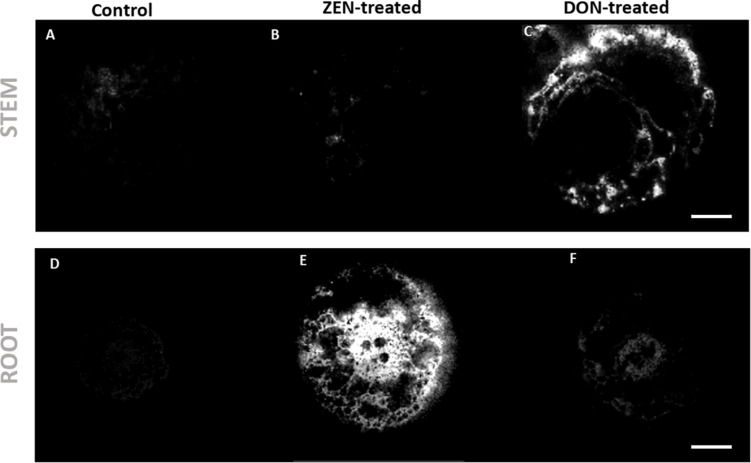
Distribution of coumaroylagmatine in root and culm samples. The hydroxycinnamic acid amide coumaroylagmatine ([M + H]^+^, *m/z* 277.1660) was found to be spread in the root of the ZEN-treated sample **(E)**, while it was localized in the outer leaf sheath of DON-treated wheat culm **(C)**. **(A–C)** Wheat culms. **(A)** Control; **(B)** ZEN-treated; **(C)** DON-treated. **(D–F)** wheat roots. **(D)** control; **(E)** ZEN-treated; **(F)** DON-treated. AP-SMALDI MS images were obtained at imaging step sizes of 7 μm for roots and 20 μm for culms and normalized to the total ion current on a 0-60% intensity scale. Scale bars: 200 μm for roots and 500 μm for culms.

On the other hand, some metabolites appear to be enhanced explicitly by each mycotoxin in the root. For instance, as depicted in Figure 3, metabolites emerging from the phenylpropanoid pathway as the hydroxycinnamic acid amide (HCAA) feruloylagmatine (Figure 3, blue) and the flavone kaempferol-rhamnoside-glucoside (Figure 3 in red) were found to be solely present in ZEN-treated root. The localization of HCAA in the leaf sheath of mycotoxin-treated culms is also consistent with their biological role associated with cell-wall strengthening and as antimicrobial agents (Zeiss et al., 2020). Furthermore, coumaroylagmatine (Figure 4), another HCAA, was co-localized with feruloylagmatine (Figure 3) only in the ZEN-treated root cortex cells. These HCAA are well-known marker compounds of a pathogen-induced tryptophan pathway (Gunnaiah and Kushalappa, 2014). These metabolites were previously localized in common wheat stem after *Fusarium graminearum* inoculum (Muroi et al., 2009; Bhandari et al., 2018; Chen et al., 2020; Zeiss et al., 2020), strengthening the hypothesis that the simple presence of ZEN may act in plants as an alerting message even before fungal colonization. This behavior manifested by ZEN could be relevant, as its higher and faster absorption by roots, along with its higher persistence and abundance in the soil where *Fusarium* grows during its saprophytic stage, may render such compound more reliable for plants in eavesdropping the presence of potential pathogens. Overall, we detected the activation of these pathways in the absence of any fungal infection, in agreement with a previous result reporting the same response activation in transgenic wheat overexpressing the multi-pathogen resistance gene Lr34 (Bucher et al., 2017).

Besides the increase of HCAA, the detection and localization of kaempferol-rhamnoside-glucoside suggest that the phenylpropanoid pathway (Watanabe et al., 2013) is influenced by ZEN accumulation.

#### Untargeted Metabolomics – Effects on Culm

As for roots, mycotoxin-treated and control culm were compared. Coumaroylagmatine accumulated in the root of ZEN-treated plantlets (Figure 4E), while it was mainly localized in the DON-treated culm (Figure 4C), suggesting a co-localization with mycotoxin occurrence. As highlighted in Figure 4C, HCAA were localized in the DON-treated culm’s outer leaf, confirming the distribution previously reported by Bhandari et al. (2018).

Other metabolites shared the same localization as coumaroylagmatine, which means accumulation in the ZEN-treated root and in the outer leaf of DON-treated culm, including feruloylagmatine, oxo-octadecadienoic acid, dihydropteroic acid (see Supplementary Tables 2, 3).

Most of these metabolites were distributed in the outer epidermis and vasculature of leaf sheath of the DON-treated culm (see Figure 4). This localization can be explained taking into consideration that, under field conditions, culm base infection is caused by *Fusarium* strains (Mudge et al., 2006; Winter et al., 2013), making this structure a primary site of pathogen migration and attack. Therefore, organ-related biological machinery to effectively counteract DON accumulation could have developed in this area due to the evolutionary advantage. As soil acts as a reservoir of future pathogens, an higher DON abundance at the root-soil interface may imply a higher risk of attack in young spikelets during wheat flowering. Thus, the reported metabolic response to environmental mycotoxin exposure may offer a better survival chance. This local defense led to the selective accumulation of dihydropteroic acid, as shown in Figure 4. This folate precursor’s production has been previously reported to be induced in *Arabidopsis* following *Pseudomonas* infection. It has been associated with enhancing salicylic acid biosynthesis and signaling (Wittek et al., 2015; Powell et al., 2017).

**FIGURE 5:**
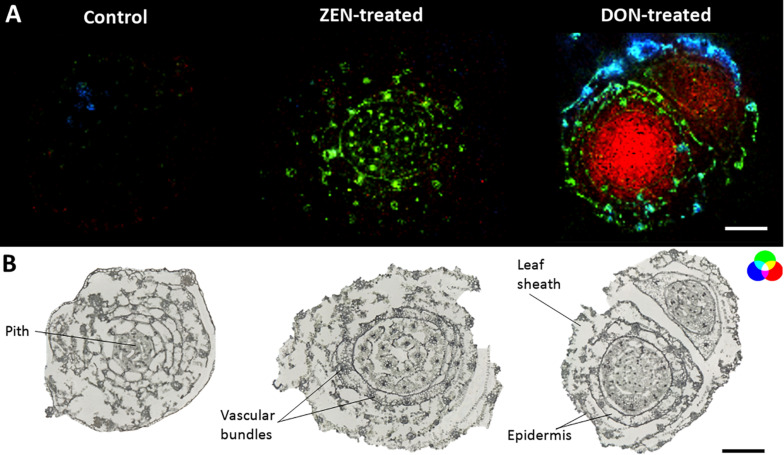
Tissue-specific mycotoxin-induced changes in wheat culms. **(A)** RGB overlay of *m/z* images of glycerophosphocholine [M + H]^+^, *m/z* 258.1101 (green), dihydropteroic acid [M + H]^+^, *m/z* 315.1199 (blue) and patuletin diglucoside [M + H]^+^, *m/z* 679.1482 (red). **(B)** Optical images of mycotoxin-treated and control stem base. AP-SMALDI MS images were obtained at 20 μm imaging step size and normalized to the total ion current on a 0-50% intensity scale. Scale bars: 500 μm.

On the other hand, a systemic defense was mediated by lipid molecules. The culm MS imaging measurement demonstrates the localization of glycerophosphocholine (Figure 5, green) in the epidermis and vascular bundles of mycotoxin-treated wheat samples. As for the glycerophospholipids annotated in the roots, the accumulation of glycerophosphocholine follows the same trend in both DON- and ZEN-treated wheat, thus representing non-specific systemic defense against xenobiotics already described for instance in *Arabidopsis* (Finnegan et al., 2016).

## Discussion

Following root uptake both DON and ZEN were absorbed from the growing medium and translocated to above-ground organs. The different behavior observed in the uptake and mobility of the two mycotoxins may be ascribable to their polarities. As plants do not handle differentially human-made and natural substances, some hypotheses for mycotoxins could be made starting from the abundant literature dealing with plant exposure to synthetic xenobiotics (Singh et al., 2016). These evidences and previous results on other *Fusarium* mycotoxins (Meng-Reiterer et al., 2016) agree that polar metabolites are more easily transportable through plant vessels, compared to less polar toxins that are hardly transported. In contrast with root uptake, translocation from underground to above-ground organs is seemingly determined by a hydrophilicity-regulated transport via xylematic tissue well described for both natural and human-made substances (Wu et al., 2013).

Having confirmed a differential handling of DON and ZEN by wheat plantlets, we proceeded to explore the spatio-temporal distributions of secondary metabolites produced in the wake of mycotoxin exposure. The aim was to evaluate if and to what extent the uptake of ZEN and DON and their accumulation in distinct plant tissues and organs may induce (or inhibit) a selective and localized metabolite production.

Being immersed in the medium containing mycotoxins, roots were the most exposed tissue and therefore the most affected. Several primary and secondary metabolites were found to be differentially accumulated in response to the treatment, including flavonoid glycosides, hydroxycinnamic acid amides, glycerophospholipids, galactolipids, and peptides (see Table 1 and Supplementary Table 1). Among these substances, some were found to be triggered by both mycotoxins, as for the images shown in Figure 2. At the same time, others were selectively accumulated only in organs from DON or ZEN treated plantlets (Figures 3, 4). These differences may be related to the distinct role that DON and ZEN play in the establishment of the disease induced in wheat by *Fusarium* spp., with DON being a well-known virulence factor (Audenaert et al., 2013) and ZEN exerting a dose-related plant-growth regulating activity (Biesaga-Kościelniak and Filek, 2010). Many overexpressed metabolites are related to the phenylpropanoid biosynthetic pathway and to phenylalanine, whose involvement in plant defense against *Fusarium* has been described previously in infected wheat. In fact, phenylpropanoids are among the most frequently reported metabolites for their potential involvement in resistance to fungal attack (Balmer et al., 2013; Doppler et al., 2019).

Furthermore, several glycerophospholipids were detected in mycotoxin-treated roots compared to the control (see Supplementary Tables 2, 3). These important signaling molecules’ production is generally related to the activation of phospholipases and membrane modifications during defense cascades (Canonne et al., 2011). In various crops, upon elicitation, phospholipases generate secondary messengers, including diacylglycerols (DG), starting a cascade that ultimately ends with accumulating phosphatidic acid. In turn, phosphatidic acid activates MAPK pathways, which in plants are related to a variety of biotic and abiotic stress stimuli, including also pathogen infection and reactive oxygen species, the latter representing a trademark in DON toxicity (Zhang and Klessig, 2001; Agrawal et al., 2003; Gupta et al., 2010). Previous studies underpinned the central role played by lipids in the mechanism of resistance toward *Fusarium* spp. (Righetti et al., 2019), while our results suggest that the activation of similar plant defense mechanisms can also be triggered by DON or ZEN alone even before the actual start of plant–fungi interaction. These findings support the hypothesis that the simple environmental exposure of certain mycotoxins may act as a trigger for wheat plantlets, prompting changes at the metabolome level toward an increased level of constitutive defense. From such a point of view, the presence of ZEN and DON in soil and their uptake in low amounts may act as an early signal of potential fungal aggression, and individuals more capable of reacting could hold an evolutionary advantage over less sensible genotypes. However, very few studies have been conducted to investigate mycotoxins’ specific role alone on the plant metabolome (Winter et al., 2013; Warth et al., 2015). Thus, their involvement in phytoanticipin and phytoalexin biosynthesis is yet to be adequately disclosed.

On the other hand, we found some metabolites enhanced explicitly by each mycotoxin in the root. This would confirm the separate role of DON and ZEN in *Triticum*–*Fusarium* interaction and imply a distinct contribution to the early alert system of wheat-related to the different absorption dynamics noticed above. Most of these metabolites are HCAA from the phenylpropanoid pathway and they were found to be solely present in ZEN-treated root. This pathway is upregulated in response to both abiotic and biotic stresses. In particular, growing literature is confirming its role in plant defense against *Fusarium* infection. The ability of HCAAs to bond cell wall polysaccharides allows the formation of linkages related to its increased resistance against fungal attack. At the same time, antioxidant and weak antifungal flavonoids contribute to a low-cost, first-line defense characteristic of phytoanticipins. Flavones, in particular, have been suggested as inhibitors of the biosynthetic step catalyzing the conversion of thricodiene to oxygenated trichothecenes like DON (Atanasova-Penichon et al., 2016).

The localization of HCAA and flavanone rhamnosyl glucoside was found to be correlated with the mycotoxin accumulation. Indeed, remarkable root segregation was observed for ZEN and its derivatives without evidencing its consistent translocation to leaves and culm. On the other hand, both metabolites, show opposite distribution when looking at the culm. Even though the fine tissue localization of DON and ZEN could not be achieved using AP-SMALDI MS, their organ partitioning was elucidated using UHPLC-HRMS. Therefore, it can be hypothesized the translocation of plant defense metabolites to the mycotoxin accumulation organs, which is the root for ZEN-treated plantlet and culm for DON-treated sample, suggesting a local “defense-on-demand response.”

The co-localization of defense metabolites and mycotoxins was previously pinpointed by Bhandari et al. (2018), who localized enniatin B in the abaxial epidermis of wheat leaf sheath together with HCCA. Simultaneously, multiple reports have confirmed, at least in different model from wheat, that priming can act at a distance: infection or exposure in a given organ can elicit resistance in a remote tissue by increasing the biosynthesis of secondary metabolites involved in plant resistance. Unfortunately, as mentioned above, the ionization of mycotoxins by MSI is quite challenging, thus we could not observe a reliable localization of masked forms of DON. However, previous HPLC-HRMS investigations (Meng-Reiterer et al., 2016; Rolli et al., 2018) have confirmed their elective accumulation in above-ground organs also after simple root exposure. Therefore, in particular, considering the translocation patterns, such long-range effects may be mediated by the presence of DON in tissues and their masked forms, particularly in above-ground organs.

The presented data offer a first insight into the possibility that DON and ZEN may be involved in the eavesdropping of *Fusarium* presence in soil and that wheat response based on secondary metabolites may operate on multiple organs with a potential interplay that involves masked mycotoxins. Therefore, present evidence underpins that priming induced by mycotoxins may warrant further investigations and suggests that the chemical crosstalk between crops and pathogenic fungi may be even more intricate than previously thought.

## Data Availability Statement

The datasets presented in this study can be found in online repositories. The names of the repository/repositories and accession number(s) can be found below: mass spectrometry imaging data that support the findings of this study have been deposited in Metaspace (https://metaspace2020.eu/datasets) with the accession code JLU Giessen_WR_Crt_92×100_7um_A26; WR_DON_121×142_7um_A26; WR_ZEN_133×164_7um_A26; WS_contorl_129×120_20um_A20_190427094307; WS_DON_133×123_20um_A20; WS_ZE N_149×123_20um_A20.

## Author Contributions

LR conceived the study, performed the sample preparations and AP-SMALDI MSI experiments, discussed the results, and wrote the manuscript. DB supported the sample preparations and AP-SMALDI MSI experiments, data analysis, and data evaluation. ER designed and performed the micropropagation experiments. ST contributed to data analysis using LipostarMSI. RB contributed to data discussion and drafting of the manuscript. CD’A and BS provided the methodology and funding, supervised the project, and critically revised the article for important intellectual content. All of the authors contributed to data discussion, according to their multidisciplinary expertise.

## Conflict of Interest

BS is a consultant and DB is a part-time employee of TransMIT GmbH, Giessen, Germany. ST is employee at Molecular Horizon srl, distributor of LipostarMSI software. The remaining authors declare that the research was conducted in the absence of any commercial or financial relationships that could be construed as a potential conflict of interest.

## Publisher’s Note

All claims expressed in this article are solely those of the authors and do not necessarily represent those of their affiliated organizations, or those of the publisher, the editors and the reviewers. Any product that may be evaluated in this article, or claim that may be made by its manufacturer, is not guaranteed or endorsed by the publisher.

## Footnotes

1 https://metaspace2020.eu

## Abbreviations

AP-SMALDI: atmospheric-pressure scanning microprobe matrix-assisted laser desorption/ionization
DG: diacylglycerols
DHB: 2,5-dihydroxybenzoic acid
DON: deoxynivalenol
DON3Glc: deoxynivalenol-3-glucoside
HCAA: hydroxycinnamic acid amide
LC: liquid chromatography
MSI: mass spectrometry imaging
MALDI: matrix-assisted laser desorption ionization
PC: phosphatidylcholine
ZEN: zearalenone.
