# Supplementary material for: Mycotoxin Uptake in Wheat — Eavesdropping Fusarium Presence for Priming Plant Defenses or a Trojan Horse to Weaken Them?
Source: Front Plant Sci. 2021 Jul 26;12:711389. doi: 10.3389/fpls.2021.711389 (PMC8350570; doi:10.3389/fpls.2021.711389)
Supplement: Supplementary file 1 [file Data_Sheet_1.pdf]

Supplementary Material

Mycotoxin uptake in wheat — Eavesdropping Fusarium presence for priming plant defenses or a trojan horse to weaken them?

Laura Righetti[1,2*], Dhaka Ram Bhandari[2], Enrico Rolli[3], Sara Tortorella[4], Renato Bruni[1], Chiara Dall'Asta[1], Bernhard Spengler[2*]

[1]Department of Food and Drug, University of Parma, Viale delle Scienze 17/A, I-43124 Parma, Italy

[2] Institute of Inorganic and Analytical Chemistry, Justus Liebig University Giessen, Heinrich-Buff-Ring 17, 35392 Giessen, Germany.

[3] Department of Chemistry, Life Sciences and Environmental Sustainability, University of Parma, Via G.P. Usberti 11/a, 43124 Parma, Italy.

[4] Molecular Horizon Srl, Via Montelino 30, 06084 Bettona, Perugia, Italy

a) ***Correspondence:**
Laura Righetti

Food and Drug Department, University of Parma, Viale delle Scienze 17/A, 43124, Parma, Italy

Email: laura.righetti@unipr.it

b) **Bernhard Spengler**

Institute of Inorganic and Analytical Chemistry, Justus Liebig University Giessen, Heinrich-Buff-Ring 17, 35392 Giessen, Germany.
Email: Bernhard.Spengler@anorg.Chemie.uni-giessen.de

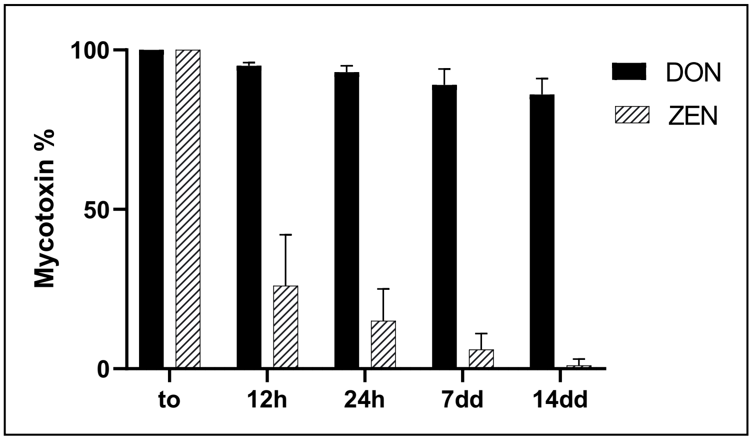

Figure S1. Residual mycotoxins in the growing medium during the growth of micropropagated plantlets of *T. durum*. Deoxynivalenol (DON) and zearalenone (ZEN) residual amounts are expressed as percentage, %, n = 3. The initial amount of toxin per treatment: 100 μg.

Note S1. Sample preparation and UHPLC-MS/MS analysis

Plant samples were separately freeze-dried for 12 h using a laboratory lyophylizator (LIO-5PDGT, 5Pascal s.r.l., Trezzano sul naviglio, Milano) and then milled. 50 mg of homogenized plant material was extracted by adding 1500 μL of a solvent mixture (acetonitrile/water/acetic acid (79:20:1, v/v) for ZEN experiment and water/methanol/acetic acid (79:20:1, v/v) for DON samples) and stirred for 90 min at 200 strokes/min on a shaker. The extract was centrifuged for 10 min at 14,000 rpm at room temperature, then 500 μL of supernatant were evaporated to dryness under nitrogen and finally resuspended in 500 μL of water/methanol (80:20, v/v) prior to LC-MS analysis. All medium samples were diluted with water/methanol (80:20, v/v) to achieve a final ratio of 1:1 (v/v), vortexed for 1 min, and then subjected to LC-MS analysis.

UHPLC Dionex Ultimate 3000 separation system coupled to a triple quadrupole mass spectrometer (TSQ Vantage; Thermo Fisher Scientific Inc., San Jose, CA, USA) equipped with an electrospray source (ESI). For the chromatographic separation, a reversed-phase C18 Kinetex column (Phenomenex, Torrance, CA, USA) with 2.10 × 100 mm and a particle size of 2.6 μm heated to 40 °C was used. A total of 2 μl of the sample extract were injected into the system; the flow rate was 0.350 ml min^{-1}. Gradient elution was performed by using 5 mmol/L ammonium acetate in water (eluent A) and methanol (eluent B), both acidified with 0.2% acetic acid. Initial conditions were set at 2% B for 1 min; then eluent B was increased to 20% in 1 min; after an isocratic step (6 min), eluent B was increased to 90% in 9 min; after a 3 min isocratic step, the system was re-equilibrated to initial conditions for 3 min. The total run time was 30 min. MS parameters: the ESI source was operated in negative-ion mode (ESI–); spray voltage 3000 V, capillary temperature 270°C, vaporiser temperature 200°C, sheath gas flow 50 units, and auxiliary gas flow 5 units. Detection of target analytes was performed using MRM mode and monitoring the $[M+CH_3COO]^-$ adduct for DON and DON3Glc, while $[M-H]^-$ was monitored for ZEN and its metabolites.

Quantification of target analytes (ZEN, ZEN14Glc, ZEN16Glc, α-and β-ZEL, DON, and DON3Glc) involved two different calibration sets. Mycotoxins content of roots and leaves was quantified by matrix-matched calibration standards prepared by dissolving an aliquot portion of the standard solution in the blank wheat extract. From the acetonitrile standards solution (1000 ng/mL) 6 diluted matrix solutions were prepared (calibration range 1−500 ng/mL). Whereas medium ZEN and DON content

was quantified by using additional matrix-matched calibration standards prepared by diluting blank medium with water/methanol (80:20, v/v) (calibration range 10−1000 ng/mL). Good linearity was obtained for all the considered mycotoxins ($R^2 > 0.99$). Quantification of selected analytes was performed employing Thermo Xcalibur 2.2.SP1 QuanBrowser software.

Note S2. MALDI matrices for mycotoxins ionization

Five matrices (DHB, CHCA, 9AA, pNA and hydrazine hydrate), the different combinations of solvents, and both polarities (see **Table S1**) were tested with the DON and ZEN standard (concentration of 10 µg/ml). CHCA (50%ACN) yielded the best signal in the dried-droplet MALDI experiment for both DON and ZEN to detect sodium adducts. Limits of detection were ∼5 pmol for DON and ∼0.3 pmol for ZEN. However, when performing on-tissue MSI, imaging experiments with these matrices ZEN and DON were not detected. Besides, a derivatization step with Girard T reagent (GirT) was tested (Barré et al., 2016). The ZEN and DON keto group's reaction gives rise to a quaternary amine (see **Figure S2 panel A**), significantly increasing ZEN and DON products' signal but not enough to observe an adequate signal for spatial distribution in the plant tissue (see **Figure S2** and **Figure S3**).

Table S1. MALDI matrices tested for mycotoxins ionization in the dried-droplet MALDI experiments. The ion species column refers to the most intense adduct detected by applying the different matrices.

Matrix	Concentration	Solvent	Polarity	Ion species	
				**DON**	**ZEN**
DHB	6 mg/mL	50% acetone	positive	$[M+Na]^+$	$[M+Na]^+$
DHB	6 mg/mL	50% ACN	positive	$[M+Na]^+$	$[M+Na]^+$
CHCA	10 mg/mL	50% acetone	positive	$[M+Na]^+$	$[M+Na]^+$
CHCA	10 mg/mL	50% ACN	positive	$[M+Na]^+$	$[M+Na]^+$
pNA	10 mg/mL	50% ACN	negative	$[M-H]^-$	$[M-H]^-$
pNA	10 mg/mL	1mmol/L CH_3COONH_4	negative	$[M-H]^-$	$[M+CH3COO]^-$
9AA	10 mg/mL	70% EtOH	positive	N.D.	$[M+H]^+$
Hydrazine hydrate	0.20%	100% H_2O	positive	$[M+Na]^+$	$[M+H]^+$
GirT derivatization	10 mg/mL	80% CH_3OH	positive	$[M+Na]^+$; DON-GirT $[M]^+$	$[M+Na]^+$; ZEN-GirT $[M]^+$

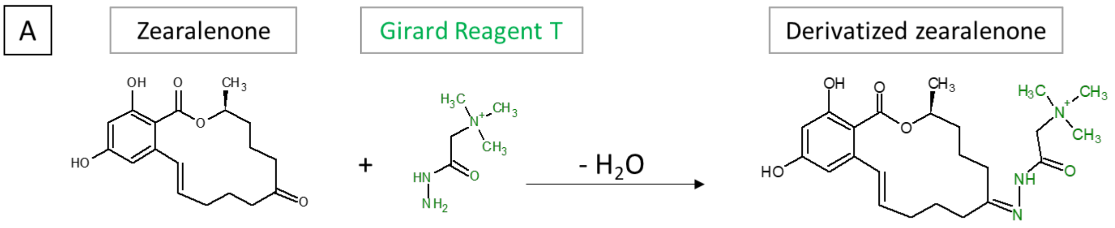
A
Zearalenone
Girard Reagent T
Derivatized zearalenone
- H2O

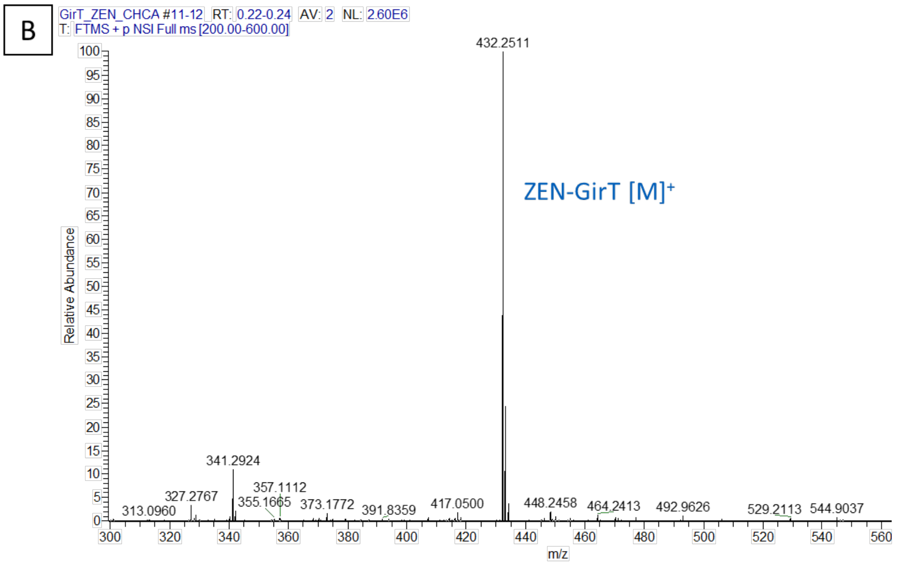
B
GirT_ZEN_CHCA #11-12 RT: 0.22-0.24 AV: 2 NL: 2.60E6
T: FTMS + p NSI Full ms [200.00-600.00]
432.2511
ZEN-GirT [M]+
341.2924
357.1112
327.2767
355.1665
373.1772
391.8359
417.0500
313.0960
448.2458
464.2413
492.9626
529.2113
544.9037
Relative Abundance
m/z

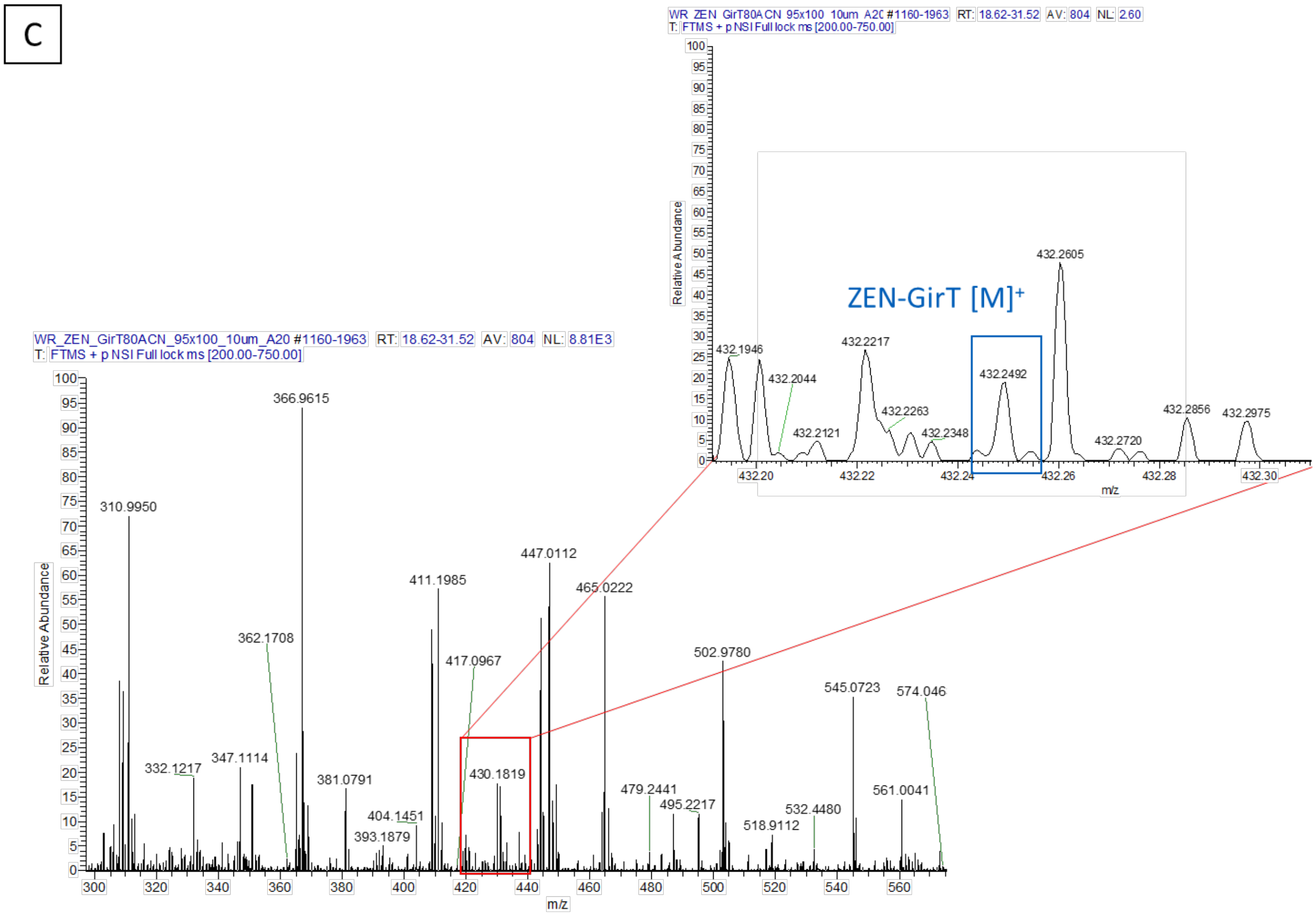

Figure S2. Result of ZEN derivatization with Girard reagent T.

(A) GirT reacts with the ZEN carbonyl functionality to form a hydrazone derivative, which incorporates a permanent positive charge. (B) The dried-droplet method was initially used to assess the formation of ZEN-GirT $[M]^+$ion at *m/z* 432.2511. (C) On-tissue derivatization led to the detection of a very low signal corresponding to ZEN-GirT $[M]^+$ ion.

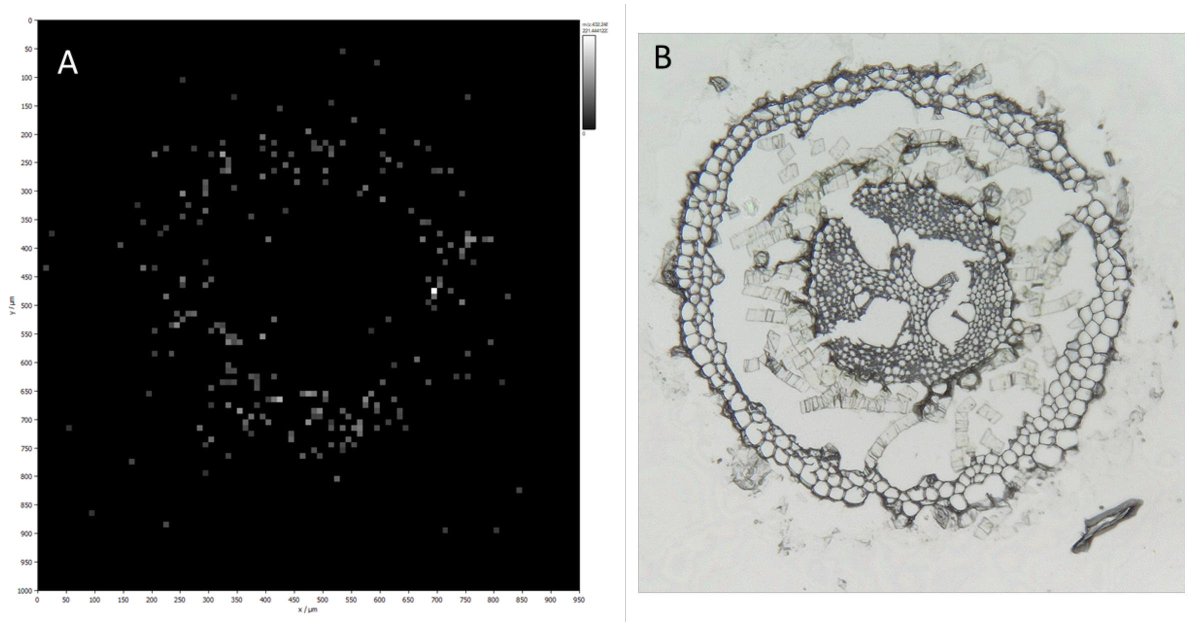

Figure S3. On-tissue derivatization of ZEN. (A) Distribution of ZEN-GirT [M]$^+$ *m/z* 432.2493 in root cortex cells and (B) its optical image. The structural integrity of some roots was damaged due to the fragility of the tissue. The AP-SMALDI-MS image of maize root was generated with 95 x 100 pixels, with a pixel size of 10 µm and an *m/z* bin width of 5 ppm.

Table S2. List of metabolites imaged in wheat roots-based and differentially accumulated in control vs. mycotoxin-treated plantlets. The reference column gives cited references for metabolites detected in previous studies on wheat or other plants. The citation details are provided in the reference list.

Compounds	Molecular formula	Adduct	Exact mass	Error ppm	Localization (treatment)	Reference
DAG (42:6)	$C_{45}H_{76}O_5$	$[M+K]^+$	735.5324	0.05	Root endodermis	
Deoxy-methoxyguanosine	$C_{11}H_{15}N_5O_5$	$[M+NH_4]^+$	315.1411	-1.62	Root cortex (control)	
Threoninyl-tryptophan	$C_{15}H_{19}N_3O_4$	$[M+NH_4]^+$	323.1714	0.56	Root cortex (control)	
Feruloylagmantine	$C_{15}H_{22}N_4O_3$	$[M+K]^+$	345.1323	1.88	Root cortex (ZEN)	(Bhandari et al., 2018)
Dihydroconiferin	$C_{16}H_{24}O_8$	$[M+H]^+$	345.1544	1.46	Root cortex (control)	(Mitchell et al., 1994)
peptide (3 aa)	$C_{24}H_{27}N_5O_6$	$[M+K]^+$	520.1593	-1.55	Root pith (control)	
peptide (3 aa)	$C_{24}H_{27}N_5O_6$	$[M+H]^+$	482.2034	-0.60	Root pith (control)	
peptide (3 aa)	$C_{21}H_{31}N_5O_6S$	$[M+H]^+$	482.2068	-0.87	Root pith (control)	
Epi-oxojasmonoyl-isoleucine	$C_{18}H_{26}NO_5$	$[M+H]^+$	337.1884	1.95	Root cortex (ZEN)	(Widemann et al., 2015)
Epicatechin(cinnamoyl-allopyranoside)	$C_{30}H_{30}O_{12}$	$[M+Na]^+$	605.1629	-0.91	Root cortex (ZEN)	(Atanasova-Penichon et al., 2016)
Dihydroxy-trimethoxy-prenyloxyflavone	$C_{23}H_{24}O_8$	$[M+Na]^+$	451.1363	-1.35	Root (ZEN)	(Atanasova-Penichon et al., 2016)
Dihydropteroic acid	$C_{14}H_{14}N_6O_3$	$[M+H]^+$	315.1200	0.36	Root (ZEN)	(Powell et al., 2017)
Hydroxy-dimethoxy-methylisoflavone - rhamnoside	$C_{24}H_{26}O_9$	$[M+Na]^+$	481.1469	-1.73	Root cortex (ZEN)	(Atanasova-Penichon et al., 2016)
Cinnamoyl-galloyl-glucopyranose	$C_{22}H_{22}O_{11}$	$[M+Na]^+$	485.1054	-1.61	Root cortex (ZEN)	(Atanasova-Penichon et al., 2016)
PC (36:3)	$C_{44}H_{82}NO_8P$	$[M+K]^+$	822.5410	-0.17	Root cortex (treated)	
MGDG (36:6)	$C_{45}H_{74}O_{10}$	$[M+K]^+$	813.4914	-0.54	Root cortex and endodermis (treated)	(Perlikowski et al., 2016)
Saponin E	$C_{42}H_{68}O_{14}$	$[M+NH_4]^+$	814.4947	-0.70	Root cortex and endodermis (treated)	
PC (34:1)	$C_{42}H_{82}NO_8P$	$[M+K]^+$	798.5410	-0.80	Root cortex and endodermis (treated)	
PC (36:6)	$C_{44}H_{76}NO_8P$	$[M+K]^+$	816.4940	0.26	Root cortex (treated)	(Rubert et al., 2017)
PI (34:5)	$C_{43}H_{73}O_{13}P$	$[M+H]^+$	829.4862	-1.02	Root cortex (ZEN)	

OKODA-PA	$C_{33}H_{57}O_{10}P$	$[M+H]^+$	645.3762	-0.29	Root epidermis (ZEN)	
Glycerophosphocoline	$C_8H_{20}NO_6P$	$[M+H]^+$	258.1101	-0.77	Root cortex (treated)	(Rubert et al., 2017)
Corchorusoside	$C_{41}H_{64}O_{19}$	$[M+Na]^+$	883.3934	1.70	Root pith (treated)	(Bhandari et al., 2018)
Oxo-octadecadiynoic acid	$C_{18}H_{26}O_3$	$[M+K]^+$	329.1514	-0.14	Root cortex (ZEN)	(Bhandari et al., 2018)
Adenosine diphosphate ribose	$C1_5H_{23}N_5O_{14}P_2$	$[M+H]^+$	560.0789	0.09	Root cortex (DON)	(Sasaki and Sugita, 1982)
Hydroxy-phenylpentanoic acid	$C_{11}H_{14}O_3$	$[M+NH_4]^+$	212.1281	-1.32	Root cortex (DON)	(Widhalm and Dudareva, 2015)
PA (36:4)	$C_{39}H_{69}O_8P$	$[M+K]^+$	735.4362	-0.05	Root pith (treated)	(Rubert et al., 2017)
PA (36:5)	$C_{39}H_{67}O_8P$	$[M+K]^+$	733.4205	0.97	Root pith (treated)	
Lyso PC (18:2)	$C_{26}H_{50}NO_7P$	$[M+K]^+$	558.2956	0.09	Root cortex (DON)	(Rubert et al., 2017)
PA (38:5)	$C_{41}H_{71}O_8P$	$[M+K]^+$	761.4518	-0.38	Root pith (treated)	
PC (34:3)	$C_{42}H_{78}NO_8P$	$[M+Na]^+$	778.5357	-1.51	Root cortex (treated)	(Rubert et al., 2017)
Ceramide phosphoimnoditols	$C_{44}H_{88}NO_{12}P$	$[M+K]^+$	892.5676	-2.08	Root cortex (treated)	

Table S3. List of metabolites imaged in wheat culm based and differentially accumulated in control vs mycotoxin-treated plantlets. The reference column gives cited references for metabolites detected in previous studies on wheat or other plants. The citation details are provided in the reference list.

Compounds	Molecular formula	Adduct	Exact mass	Error ppm	Localization (treatment)	Reference
Trihydroxy-methylacridone	$C_{14}H_{11}NO_4$	$[M+H]^+$	258.0761	-0.06	Epidermis and vb (treated)	
Glycerophosphocoline	$C_8H_{20}NO_6P$	$[M+K]^+$	296.0660	0.62	Epidermis and vb (treated)	
Peonidin	$C_{16}H_{13}O_6$	$[M+H]^+$	302.0785	-0.37	Stem pith (DON)	(Chen et al., 2013)
Feruloylagmantine	$C_{15}H_{22}N_4O_3$	$[M+H]^+$	307.1765	0.87	Leaf sheath (DON)	(Bhandari et al., 2018)
Avenic acid A	$C_{12}H_{22}N_2O_8$	$[M+Na]^+$	345.1268	-0.76	Leaf sheath (DON)	(Neelam et al., 2012)
peptide (4 aa)	$C_{16}H_{30}N_4O_5S$	$[M+Na]^+$	413.1829	1.93	Leaf sheath (DON)	
peptide (4 aa)	$C_{16}H_{30}N_4O_5S_2$	$[M+H]^+$	423.1730	0.80	Stem pith (DON)	
Oxo-octadecadiynoic acid	$C_{18}H_{26}O_3$	$[M+K]^+$	329.1514	-0.14	Leaf sheath (DON)	(Bhandari et al., 2018)
Cellobiosylglucose	$C_{18}H_{32}O_{16}$	$[M+Na]^+$	527.1583	-1.41	Vb (DON)	(Bhandari et al., 2018)
Patuletin-glucoside	$C_{22}H_{22}O_{13}$	$[M+Na]^+$	517.0953	0.31	Leaves (DON)	
Myricetin -galloylrhamnoside	$C_{29}H_{26}O_{16}$	$[M+Na]^+$	653.1113	-0.14	Leaves (DON)	(Atanasova-Penichon et al., 2016)
Hydroxypachyrrhizone	$C_{20}H_{14}O_8$	$[M+H]^+$	383.0761	-0.41	Leaves (DON)	(Chen et al., 2013)
DG (34:4)	$C_{37}H_{64}O_5$	$[M+H-H2O]^+$	571.4721	0.15	Leaf sheath (ZEN)	(Rubert et al., 2017)
PI (38:8)	$C_{47}H_{75}O_{13}P$	$[M+K]^+$	917.4577	0.31	Leaf sheath (ZEN)	
PE (12:0)	$C_{17}H_{34}NO_8P$	$[M+H]^+$	412.2095	0.68	Stem pith (ZEN)	
PC (25:0 (CHO))	$C_{33}H_{64}NO_9P$	$[M+H]^+$	650.4391	0.22	Vb (DON)	

Table S4. Analytical details on accurate mass, detected adduct, formula and error ppm of those metabolites discussed throughout the manuscript.

Class	Compounds	Molecular formula	Adduct	Exact mass	Error ppm
Amino acids, peptides, and analogs	Tryptophyl-alanine	$C_{14}H_{17}N_3O_3$	$[M+NH_4]^+$	293.1608	0.74
Glycerophospholipid	PC (34:3)	$C_{42}H_{78}NO_8P$	$[M+K]^+$	794.5097	-0.3
Galactolipid	DGDG (34:2)	$C_{49}H_{88}O_{15}$	$[M+K]^+$	955.5755	1.06
Hydroxycinnamic acids amides	Feruloylagmantine	$C_{15}H_{22}N_4O_3$	$[M+H]^+$	307.1765	-0.11
Flavonoid glycosides	Kaempferol-rhamnoside-glucoside	$C_{27}H_{30}O_{15}$	$[M+Na]^+$	617.1477	-0.18
Diacylglycerols	DG (42:5)	$C_{45}H_{78}O_5$	$[M+K]^+$	737.5481	-0.02
Hydroxycinnamic acids amides	Coumaroylagmatine	$C_{14}H_{20}N_4O_2$	$[M+H]^+$	277.1659	-0.35
Glycerophospholipid	Glycerophosphocoline	$C_8H_{20}NO_6P$	$[M+H]^+$	258.1101	-0.77
Flavonoid glycosides	Patuletin diglucoside	$C_{28}H_{32}O_{18}$	$[M+Na]^+$	679.1481	-0.17
Pteridines and derivatives	Dihydropteroic acid	$C_{14}H_{14}N_6O_3$	$[M+H]^+$	315.12	0.36

References

Atanasova-Penichon, V., Barreau, C., and Richard-Forget, F. (2016). Antioxidant secondary metabolites in cereals: Potential involvement in resistance to Fusarium and mycotoxin accumulation. *Front. Microbiol.* 7, 1–16. doi:10.3389/fmicb.2016.00566.

Barré, F. P. Y., Flinders, B., Garcia, J. P., Jansen, I., Huizing, L. R. S., Porta, T., et al. (2016). Derivatization strategies for the detection of triamcinolone acetonide in cartilage by using matrix-assisted laser desorption/ionization mass spectrometry imaging. *Anal. Chem.* 88. doi:10.1021/acs.analchem.6b02491.

Bhandari, D. R., Wang, Q., Li, B., Friedt, W., Römpp, A., Spengler, B., et al. (2018). Histology-guided high-resolution AP-SMALDI mass spectrometry imaging of wheat-Fusarium graminearum interaction at the root–shoot junction. *Plant Methods* 14, 103. doi:10.1186/s13007-018-0368-6.

Chen, W., Müller, D., Richling, E., and Wink, M. (2013). Anthocyanin-rich purple wheat prolongs the life span of Caenorhabditis elegans probably by activating the DAF-16/FOXO transcription factor. *J. Agric. Food Chem.* 61, 3047–3053. doi:10.1021/jf3054643.

Mitchell, H. J., Hall, J. L., and Barber, M. S. (1994). Elicitor-induced cinnamyl alcohol dehydrogenase activity in lignifying wheat (Triticum aestivum L.) leaves. *Plant Physiol.* 104, 551–556. doi:10.1104/pp.104.2.551.

Neelam, K., Rawat, N., Tiwari, V., Prasad, R., Tripathi, S., Randhawa, G., et al. (2012). Evaluation and identification of wheat-Aegilops addition lines controlling high grain iron and zinc

concentration and mugineic acid production. *Cereal Res. Commun.* doi:10.1556/CRC.40.2012.1.7.

Perlikowski, D., Kierszniowska, S., Sawikowska, A., Krajewski, P., Rapacz, M., Eckhardt, Ä., et al. (2016). Remodeling of leaf cellular glycerolipid composition under drought and re-hydration conditions in grasses from the Lolium-Festuca complex. *Front. Plant Sci.* 7. doi:10.3389/fpls.2016.01027.

Powell, J. J., Fitzgerald, T. L., Stiller, J., Berkman, P. J., Gardiner, D. M., Manners, J. M., et al. (2017). The defence-associated transcriptome of hexaploid wheat displays homoeolog expression and induction bias. *Plant Biotechnol. J.* 15, 533–543. doi:10.1111/pbi.12651.

Rubert, J., Righetti, L., Stranska-Zachariasova, M., Dzuman, Z., Chrpova, J., Dall'Asta, C., et al. (2017). Untargeted metabolomics based on ultra-high-performance liquid chromatography–high-resolution mass spectrometry merged with chemometrics: A new predictable tool for an early detection of mycotoxins. *Food Chem.* 224, 423–431. doi:10.1016/j.foodchem.2016.11.132.

Sasaki, K., and Sugita, M. (1982). Relationship between Poly(Adenosine 5′-diphosphate-ribose) Synthesis and Transcriptional Activity in Wheat Embryo Chromatin. *Plant Physiol.* doi:10.1104/pp.69.2.543.

Widemann, E., Heitz, T., Miesch, L., Miesch, M., Heinrich, C., Pinot, F., et al. (2015). Identification of the 12-oxojasmonoyl-isoleucine, a new intermediate of jasmonate metabolism in Arabidopsis, by combining chemical derivatization and LC–MS/MS analysis. *Metabolomics* 11, 991–997. doi:10.1007/s11306-014-0754-7.

Widhalm, J. R., and Dudareva, N. (2015). A familiar ring to it: Biosynthesis of plant benzoic acids. *Mol. Plant*. doi:10.1016/j.molp.2014.12.001.
